# Prenatal Progestin Exposure Is Associated With Autism Spectrum Disorders

**DOI:** 10.3389/fpsyt.2018.00611

**Published:** 2018-11-19

**Authors:** Ling Li, Min Li, Jianping Lu, Xiaohu Ge, Weiguo Xie, Zichen Wang, Xiaoling Li, Chao Li, Xiaoyan Wang, Yan Han, Yifei Wang, Liyan Zhong, Wei Xiang, Xiaodong Huang, Haijia Chen, Paul Yao

**Affiliations:** ^1^Department of Pediatrics, Hainan Maternal and Child Health Hospital, Haikou, China; ^2^Institute of Rehabilitation Center, Tongren Hospital of Wuhan University, Wuhan, China; ^3^Department of Child Psychiatry, Kangning Hospital of Shenzhen, Shenzhen, China; ^4^SALIAI Stem Cell Institute of Guangdong, Guangzhou SALIAI Stem Cell Science and Technology Co. LTD., Guangzhou, China

**Keywords:** Autism spectrum disorders, estrogen receptor β, prenatal exposure, progestin, rat, Zebrafish

## Abstract

We have previously reported that prenatal progestin exposure induces autism-like behavior in offspring through ERβ (estrogen receptor β) suppression in the brain, indicating that progestin may induce autism spectrum disorders (ASD). In this study, we aim to investigate whether prenatal progestin exposure is associated with ASD. A population-based case-control epidemiology study was conducted in Hainan province of China. The ASD children were first screened with the Autism Behavior Checklist (ABC) questionnaire, and then diagnosed by clinical professionals using the ASD diagnosis criteria found in the Diagnostic and Statistical Manual of Mental Disorders, Fifth Edition (DSM-V). Eventually, 235 cases were identified as ASD from 37863 children aged 0–6 years old, and 682 matched control subjects with typically developing children were selected for the analysis of potential impact factors on ASD prevalence using multivariate logistic regression. Our data show that the ASD prevalence rate in Hainan was 0.62% with a boy:girl ratio of 5.4:1. Interestingly, we found that the following factors were strongly associated with ASD prevalence: use of progestin to prevent threatened abortion, use of progestin contraceptives at the time of conception, and prenatal consumption of progestin-contaminated seafood during the first trimester of pregnancy. All the above factors were directly or indirectly involved with prenatal progestin exposure. Additionally, we conducted *in vivo* experiments in rats to further confirm our findings. Either endogenous (progesterone) or synthetic progestin (norethindrone)-treated seafood zebrafish were used to feed pregnant dams, and the subsequent offspring showed autism-like behavior, which further demonstrated that prenatal progestin exposure may induce ASD. We conclude that prenatal progestin exposure may be associated with ASD development.

## Introduction

Autism spectrum disorders (ASD) is a neurodevelopmental disorder characterized by deficits in social interaction and communication in addition to repetitive and stereotyped behaviors (1). Epidemiological studies have revealed a worldwide rising trend in ASD prevalence in the past decades, with the most recent reported ratio of 1:68 (2, 3). ASD has become the most common disease in childhood disability, having severe impacts on families and society. Recent studies have identified many risk factors for ASD prevalence, including genetics/epigenetics, genders and environmental factors (2, 4), although the exact etiology remains unknown (5).

Epidemiology study shows that maternal hormonal interventions is a potential risk factor for ASD development (6–8). ASD patients have elevated steroidogenic activity (9), and cholesterol metabolism, various steroid abnormalities, and vitamin D metabolism are involved in ASD development (10, 11). In addition, oral contraceptives have been reported to be associated with ASD prevalence (12, 13), as progesterone suppresses ERβ expression (14, 15). Progestin can also regulate neurogenic responses (16) and impair cognitive response during development (17) by down-regulating ERβ expression (18, 19). It has been reported that ERβ dysregulation in the brain has been found in ASD subjects (20), and, very interestingly, we have recently reported that prenatal exposure of either levonorgestrel (LNG) (19) or any of the other 8 kinds of synthetic progestins (21) induces autism-like behavior in offspring through ERβ suppression in rats (19, 21). This indicates that prenatal progestin exposure, together with subsequent maternal hormone dysfunction, may result in ERβ downregulation and contribute to ASD development (14, 15). We hypothesize that prenatal progestin exposure is a risk factor for ASD development through ERβ downregulation in the brain.

In an effort to find epidemiological evidence for the potential association between prenatal progestin exposure and ASD, a population-based case-control epidemiology study was conducted in Hainan province of China in order to analyze potential impact factors on ASD development. We found that the following 3 factors were strongly associated with ASD prevalence: use of progestin to prevent threatened abortion, use of progestin contraceptives at the time of conception, and prenatal consumption of progestin-contaminated seafood during the first trimester of pregnancy. Further, *in vivo* experiments in rats showed that prenatal consumption of progestin-contaminated seafood zebrafish suppressed ERβ expression in the brain and induced autism-like behavior in the offspring. Given that prenatal progestin exposure could induce autism-like behavior in offspring as we reported previously (19), our current study on epidemiological data and progestin-contaminated seafood strengthens our conclusion that prenatal progestin exposure may be associated with ASD development.

## Materials and methods

### Participants for evaluation of ASD risk factors

This human subjects study was approved by the Human Subjects Institutional Review Board from Hainan Maternal and Child Health Hospital. Children under 6 years old in Hainan province participated in this investigation along with their matched parents, and informed written consents were obtained from all the participants. Participating children were born during the period of Aug. 31th, 2007–Sep. 1st, 2014, and had lived in their community for at least 1 year. Screening questionnaires were filled out by parents of the participants during the period of Sep–Nov 2015, while clinical diagnosis was conducted during the period of May–Sep 2016. Stratified cluster sampling methods were used in a total of 18 cities and counties in Hainan province, which were further classified into the following 4 types: City, 1st Type County; 2nd Type County and 3rd Type County according to their geographical location, economic development level, and infant mortality rate. In the city area, average income is the highest and infant mortality rate is the lowest, and the counties are further separated into 1st, 2nd, and 3rd Type with decreasing levels of average income. It was ensured that the number of sampled subjects in each class was appropriate to the total populations with good representativeness.

### Autistic traits

All the participants or their parents were required to complete an autism spectrum quotient (AQ) form. The participants with ≥80 intelligence quotient score (full-scale IQ) were included, but children who had co-morbid psychiatric symptoms or histories of ADHD (Attention Deficit/Hyperactivity Disorder), epilepsy, depression, or brain injury were ruled out (22). All the participants were first evaluated using the Autism Behavior Checklist (ABC), which is widely used as an ASD screening tool in China (23). This checklist is suitable for screening of populations aged between 8 months to 28 years old. ABC scores >53 points indicate high probability of ASD symptoms, while ABC scores >67 points indicate obvious ASD symptoms (22, 24, 25).

### ASD diagnosis

ASD diagnosis was based on several clinical assessments, which included clinical observation, cognitive testing, and child development history by a multidisciplinary team. This was further confirmed by licensed clinical psychologists and psychiatrists in Hainan Maternal and Child Health Hospital using the ASD diagnosis criteria in the Diagnostic and Statistical Manual of Mental Disorders, Fifth Edition (DSM-5). The positive candidates had at least 3 abnormalities in social communication/interaction and 2 abnormalities in restricted and repetitive behaviors (22, 26).

### Evaluation of potential ASD risk factors using case-control study

We investigated the potential association between prenatal progestin exposure and ASD development. Two hundred and thirty five autistic children were diagnosed and confirmed in total, and both autistic children and their parents were included for this survey. Each individual autistic child was rigorously matched with 3 control subjects of typically developing (TD) children, and eventually 698 individuals were selected after exclusion of outliers. Candidates with any types of psychiatric disorders were excluded based on the Kiddie-SADS-Present and Lifetime Version (K-SADS-PL) (23). Both autistic and TD children were assessed for verbal IQ (intelligence quotient), performance IQ and full-scale IQ using the Wechsler Intelligence Scale for Children IV (WISC-IV) (22, 23). As a result, the autistic and TD did not differ significantly in age, sex, total IQ, performance IQ and verbal IQ. Eventually, the children's mothers were requested to fill a questionnaire, which included their basic information (such as name, age, gender, home address, phone number, etc.) and the following risk factors: family history of mental diseases, pre-term birth (< 37 weeks), birth asphyxia; paternal age, maternal age, paternal personality, maternal personality, major stressful life event (including bereavement, divorce and job loss) during the first trimester of pregnancy (7, 27), paternal smoking and areca chewing history, maternal abortion history, use of progestin to prevent threatened abortion during the first trimester of pregnancy, use of progestin contraceptives at the time of conception, and prenatal consumption of progestin-contaminated seafood during the first trimester of pregnancy. The potential impact of risk factors on ASD prevalence was analyzed using chi-square (χ^2^) test, followed by multivariate logistic regression analysis together with the calculation of odds ratio (OR) and 95% confidence interval (CI).

### *In vivo* experiments for zebrafish

The animal protocol conformed to US NIH guidelines (Guide for the Care and Use of Laboratory Animals, No. 85–23, revised 1996), and was reviewed and approved by the Institutional Animal Care and Use Committee from Wuhan University and Hainan Maternal and Child Health Hospital (19). Norethindrone (NET, #1469005) and progesterone (P4, #P0130) were obtained from Sigma. Adult male zebrafish (10 months old) were obtained from a local supplier and randomly placed into 10 L stainless steel tanks in aerated water (10 fish/tank). The experimental setup consisted of a solvent control (CTL, 0.01% DMSO), 10,00 ng/L, and 50,00ng/L of P4 and NET exposure groups. Each group consisted of 5 replicates. O_2_ concentration (>70%) and pH (6.7–7.2) were measured continuously and kept stable to ensure water quality, and temperature was kept constant at 27 ± 1°C with the light:dark cycle of 14:10 h. All fish were exposed to the chemicals for 1 month to achieve the most significant long-term NET/P4 exposure. The fish were maintained in a continuous flow through the system to ensure a complete change of reconstituted water every 8 h. During the experimental process, individuals with significant abnormalities in appearance and mortality were recorded and removed, and food was provided as in the cultivation. The protocol was conducted according to the Organization for Economic Co-operation and Development (OECD) Guideline 204. At the end of exposure, zebrafish were euthanized by submersion in ice water (5 parts ice/1 part water, 0–4°C) for at least 10 min following cessation of opercular movement. All the exposed fish from each of the 5 replicates were dissected immediately to isolate the brain to be stored at −80°C for subsequent RNA extraction and mRNA analysis by real time PCR (18, 28, 29).

### Chemical analysis

Concentrations of NET and P4 in exposed water were analyzed using rapid resolution liquid chromatography-tandem mass spectrometry (RRLC-MS/MS) method with electrospray ionization (ESI) as described previously with minor modifications (30, 31). The water samples containing either NET or P4 (500 mL) were collected at the exposure time of 0 and 24 h (prior to water renewal) from all replicate beakers. The water samples were extracted by solid-phase extraction using BAKERBOND™ spe Octadecyl (C18) Disposable Extraction Columns (#7020-01, Avantor). After loading, the cartridges were vacuum-dried for 1 h and then eluted with 10 mL ethyl acetate. The extracts were dried under nitrogen and dissolved in 1 mL methanol for analysis by RRLC-MS/MS (Agilent 1200 LC-Agilent 6460 QQQ). The limits of detection (LOD) for NET and P4 were 0.05 and 0.10 ng/L, respectively (30, 31).

### *In vivo* experiments for rats

Sprague Dawley rats (Wuhan, China) were maintained under standard 12h light/dark cycles and given *ad libitum* access to food and water. Adult (3 months old) female Sprague Dawley rats were monitored for estrous cycles with daily vaginal smears. Only rats with at least two regular 4–5 days estrous cycles were included in the studies. The females were caged with proven males, and pregnancy was verified by observation of a sperm plug, which was designated as day 0 of pregnancy (19). The progestin-exposed zebrafish were sacrificed and dried, then grounded into powder and mixed with normal chew in the ratio of 1:1 (g/g); this mixture was used to feed the pregnant dams. Dams were randomly assigned into 3 groups and fed with different concentrations of progestin (NET or P4)-exposed zebrafish food continuously from day 1 until pup delivery for ~21 days. Group 1: 0 ng/L progestin (P4 or NET)-exposed zebrafish food; Group 2: 1,000 ng/L progestin-exposed zebrafish food; Group 3: 5,000 ng/L progestin-exposed zebrafish food. The subsequent offspring were used for autism-like behavior testing at 10 weeks old. After that, the offspring were sacrificed, and the amygdala was isolated, flash frozen in dry ice, and then stored in a −80°C freezer for the analysis of gene expression (19).

### Animal behavior tests

The animal behavior tests were carried out when the offspring were 10 weeks old. Female offspring were tested in the diestrus phase, which was confirmed by vaginal smears. Autism-like behavior was evaluated using the marbles burying test (MBT), social interaction (SI) test and elevated plus maze (EPM) test (19, 32, 33). In this study, the marbles burying test (MBT) and elevated plus maze (EPM) test were used for evaluation of autism-like behavior even though they are traditionally used for anxiety-like behavior tests; this is because many studies have shown that social anxiety is sometimes associated with autism-like behavior in rodents (34, 35), especially when ERβ is involved (36, 37).

#### Marbles burying test (MBT)

In brief, each rat was placed in a clean cage (35 × 23 × 19 cm^3^) filled with wood chip bedding to a depth of 5 cm containing 20 colored glass marbles (1 cm diameter) placed in a 5 × 4 arrangement. The number of marbles buried (>50% covered by bedding material) after 30 min was hand-scored by the experimenter (19, 32, 38).

#### Social interaction (SI) test

In short, the subjects (Test and Stranger) were separately habituated to the arena for 5 min before the test. During each test, the rats were placed into the apparatus over a period of 20 min. The time spent following, mounting, grooming, and sniffing any body parts of the other rat was taken as an indicator of social engagement, and the social interaction time was collected and analyzed using EthoVision XT animal tracking software (Noldus, USA) (39). The animal used as the “Stranger” was used only once, and was a Sprague Dawley rat of the same gender, weight, and age, with no previous contact with the test rats (19, 32, 38).

#### Elevated plus maze (EPM)

To investigate the presence of anxiety-like behavior in male and female offspring, the EPM test, a well-established rodent model used to characterize anxiety-like behavior, was performed. The Elevated Plus Maze Package with IR Beam Detection for Rat (Cat #: MED-ELVM-1R) was obtained from Med Associates Inc. The maze comprised two open and two closed arms. Dual sensors at the entrance of each goal runway allow software to differentiate between runway exploration and entrance, resulting in more accurate position detection. The rats were placed in the junction area and their movements were measured for 5 min using infrared beams installed on each arm and automatically registered by the MED-PC software (Cat #: SOF-735, Med Associates) for further analysis (19, 40).

### RT reaction and real-time quantitative PCR

Total RNA from the amygdala was extracted using the RNeasy Micro Kit (Qiagen), and the RNA was reverse transcribed using an Omniscript RT kit (Qiagen). All the primers were designed using Primer 3 Plus software with the Tm at 60°C, primer size of 21 bp, and the product length in the range of 140–160 bp (see Table 1). The primers were validated with the amplification efficiency in the range of 1.9–2.1, and the amplified products were confirmed with agarose gel. The real-time quantitative PCR was run on iCycleriQ (Bio-Rad) with the Quantitect SYBR green PCR kit (Qiagen). The PCR was performed by denaturation at 95°C for 8 min, followed by 45 cycles of denaturation at 95°C, annealing at 60°C, and extension at 72°C for 10 s, respectively. one microliter of each cDNA was used to measure target genes. The β-actin was used as the housekeeping gene for transcript normalization, and the mean values were used to calculate relative transcript levels with the ^ΔΔ^CT method per instructions from Qiagen. In brief, the amplified transcripts were quantified by the comparative threshold cycle method using β-actin as a normalizer. Fold changes in gene mRNA expression were calculated as 2^−−ΔΔ*CT*^ with CT = threshold cycle, ΔCT = CT(target gene)-CT(β-actin), and the ΔΔCT = ΔCT(experimental)-ΔCT (reference) (41).

**Table 1:** Sequences of primers for the real time quantitative PCR (qPCR).

**Gene**	**Species**	**Forward primer (5^′^ → 3^′^)**	**Reverse primer (5^′^ → 3^′^)**
β-actin	Rat	ttccttcctgggtatggaatc	Cttctgcatcctgtcagcaat
ERβ	Rat	tcagcatgaagtgcaaaaatg	Ggttctgggagctctctttgt
ERRα	Rat	cagtgggaagctagtgctcag	Ggacagctgtactcgatgctc
SOD2	Rat	caactcaggttgctcttcagc	Ctcaaaagacccaaagtcacg
β-actin	Zebrafish	ggacctgtatgccaacacagt	Accgatccagacggagtattt
ERβ	Zebrafish	ctttattttggccacctcaca	Cttcaccagtggtttgctgtt
ERRα	Zebrafish	aaagaaggagttcgcttggac	Ccagcagatgagacacaatga
SOD2	Zebrafish	taaagcgtgactttggctcat	Caaagggtcttggttagcaca

### Statistical analysis

The data was given as mean ± *SD*. One-way ANOVA followed by the Turkey–Kramer test was used to compare multiple group differences. Comparisons were made on each variable using χ^2^ tests, and student *t*-tests was used for continuous variables. Multivariate logistic regression was used for analysis of the impact of variables with the calculation of odds ratio (OR) and 95% confidence intervals (CI). Furthermore, a factorial design ANOVA was used for the analysis of the basic experimental design following a full-factorial 3 × 2 (prenatal treatment × sex) design study, and the Fisher's Least Significant Difference (LSD) test was conducted for multiple comparison when there was a significant effect on prenatal treatment. The data were analyzed using SPSS 22 software, and a *P* value of < 0.05 was considered significant (19, 42).

## Results

### General information for ASD participants

The survey investigated 38,267 children in a total of 18 cities and counties in Hainan province; excluding 405 over-aged children, the remaining valid participants totaled 37,862, which included 20,824 boys and 17,038 girls with a male/female ratio of 1.2/1. The participants of < 1 year old totaled 6,401 (16.91%), the participants of 1–2 years old totaled 6,619 (17.48%), the participants of 2–3 years old totaled 6,451 (17.03%), the participants of 3–4 years old totaled 5,925 (15.65%), the participants of 4–5 years old totaled 5,468 (14.44%), the participants of 5–6 years old totaled 4,436 (11.72%), and the participants of 6–7 years old totaled 2,562 (6.77%). The demographic distribution data for each region are shown in Table 2. Finally, 235 cases of ASD were identified in total; and the lowest score on the ABC scale was 52 and the mean score was 81.4 ± 23.8. The prevalence rate of ASD in Hainan Province is 0.62%; the prevalence rate of ASD in the City area was 1.37%, along with the prevalence rate in 1st-Type County of 0.47%, 2nd-Type County of 0.34% and 3rd-Type County of 0.38%. χ^2^ test showed that there was significant regional difference in ASD prevalence (χ^2^ = 114.77, *P* < 0.01). Our results suggest that well-developed regions with higher incomes had a higher ASD prevalence than less developed regions.

**Table 2:** General information for the participants in the population-based ASD study.

	**City area**	**1st-Type county**	**2nd-Type county**	**3rd-Type county**	**χ^2^ value**	***P* value**	**Total**
Theoretic Survey Number	10,230	4,580	15130	10060	76.031 (df = 9)	0.000	40,000
Actual Survey Number	9,459	4,543	14281	9984			38,267
Over-aged Number^*^	35	63	181	126			405
Validated No (%)	9,424 (99.63%)	4,480 (98.61%)	14,100 (98.73%)	9,858 (98.74%)			37,862 (98.94%)
Male (%)	5,263 (55.85%)	2,469 (55.11%)	7,731 (54.83%)	5,361 (54.38%)	1.286 (df = 3)	0.732	20,824 (55.00%)
Female (%)	4,161 (44.15%)	2,011 (44.89%)	6369 (45.17%)	4,497 (45.62%)			17,038 (45.00%)
ASD cases (%)	129 (1.37%)	21 (0.47%)	48 (0.34%)	37 (0.38%)	114.772 (df = 3)	0.000	235 (0.62%)
TD subjects (%)	9,295 (98.63%)	4,459 (99.53%)	140,529 (99.66%)	9,821 (99.62%)			37,627 (99.38%)

* *Over-aged Number indicates the number of children aged over 83 months or older than 6 years 11 months of age. City area includes Haikou and Sanya City; 1st-type Counties include Qionghai and Wenchang; 2nd-type Counties include Wanning, Ding'an, Tunchang, Chengmai, Lingao and Danzhou; 3rd-type Counties include Lingshui, Ledong, Baoting, Wuzhishan, Qiongzhong, Dongfang, Changjiang and Baisha. df, degrees of freedom; TD, typically developing*.

### Contribution of gender and age on ASD prevalence

The ASD prevalence rate for children 0–6 years old in Hainan province was 0.62%, with 0.99% for boys and 0.17% for girls. The ASD prevalence rate for boys was significantly higher than that of girls, with the boy:girl ratio of 5.8:1. The χ^2^ test showed that the rate of ASD prevalence in boys and girls in Hainan province was significantly different (χ^2^ = 101.91, *P* < 0.01). Furthermore, the ASD prevalence rate of the group aged < 3 years old was 0.17%, group aged 3–4 years old was 0.71%, group aged 4–5 years old was 0.84%, group aged 5–6 years was 0.91%, and the group aged >6 years old was 2.89%. The χ^2^ test showed that the ASD prevalence showed an increasing trend with increasing of ages (χ^2^ = 222.39, *P* < 0.01); see detailed information in Table 3.

**Table 3:** Potential impacts of genders and ages on ASD prevalence.

	**ASD**	**Control**	**χ*^2^* value**	***P* value**
Male	206 (0.99%)	20,618 (99.01%)	101.912 (df = 1)	< 0.001
Female	29 (0.17%)	17,009 (99.83%)	
< 3 years	33 (0.17%)	19,438 (99.83%)	288.618 (df = 4)	< 0.001
3–4 years	42 (0.71%)	5,883 (99.29%)	
4–5 years	46 (0.84%)	5,442 (99.16%)	
5–6 years	40 (0.91%)	4,345 (99.09%)	
>6 years	74 (2.89%)	2,488 (97.11%)	
Total	235	37,627

*< 3 years refer to the age of < 36 months, 3–4 years refer to the age of 37–48 months; 4–5 years refer to the age of 49–60 months; 5–6 years refer to the age of 61–72 months; >6 years refer to the age of 73–83 months. df: degrees of freedom*.

### Evaluation of potential ASD risk factors using case-control study

We investigated the potential impact of risk factors on ASD prevalence in Hainan province using population-based case-control study. In total, 263 cases of ASD children from a participating population of 37,862 were identified, and another 682 typically developing (TD) subjects were collected to achieve around 3-matched control subjects for each ASD case after exclusion of outliers. We performed rigorous matching between ASD cases and control subjects to achieve no difference in age, sex, total IQ, performance IQ and verbal IQ (see details in Table 4). We further performed the chi square (χ^2^) test for each factor, and did not observe any significant impact for the following potential risk factors: family history of mental diseases, pre-term birth (< 37 weeks), birth asphyxia, paternal age, paternal personality, maternal personality, major stressful life event during the first trimester of pregnancy, paternal smoking and areca chewing history, and maternal abortion history. On the other hand, we found significant impacts for the factors of maternal age, use of progestin to prevent threatened abortion during the first trimester of pregnancy, use of progestin contraceptives at the time of conception, and prenatal consumption of progestin-contaminated seafood during the first trimester of pregnancy (see details in Table 5).

**Table 4:** Participant characteristics in ASD case-control study (Mean ± *SD*).

	**Autistic case group (*n* = 235)**	**TD control group (*n* = 682)**	**Statistic (t or χ^2^ value)**	***P* value**
Age (years)	5.26 ± 1.30	5.34 ± 1.14	0.842 (*t*-Test)	0.400
Sex (m/f)	172/31	89/16	0.000 (χ^2^ test, df = 1)	0.994
Full scale IQ	102.35 ± 14.45	103.86 ± 10.59	1.476 (*t*-Test)	0.141
Performance IQ	103.66 ± 15.53	104.78 ± 8.69	1.056 (*t*-Test)	0.292
Verbal IQ	103.09 ± 14.10	104.22 ± 10.43	1.127 (*t*-Test)	0.260

*m/f, male/female ratio; df, degrees of freedom; TD, typically developing; IQ, intelligence quotient*.

**Table 5:** The potential impact factors for ASD in population-based case-control study.

**Impact factors**	**No. of cases (*n* = 235)**	**No. of controls (*n* = 682)**	**Statistic (χ^2^ value)**	***P* value**
Family History of Mental Diseases	23	56	0.551(df = 1)	0.458
Preterm birth (< 37 weeks)	25	57	1.1161(df = 1)	0.291
Birth Asphyxia	34	74	2.2011(df = 1)	0.138
Paternal Age			0.8841(df = 2)	0.643
< 20 years	12	36	
21–34 years	184	550	
>34 years	39	96	
Maternal Age (year)			10.8691(df = 2)	0.004^*^
< 20 years	21	47	
21-34 years	145	498	
>34 years	69	137	
Paternal Personality			0.4021(df = 2)	0.818
Extroverted	30	79	
Middle	150	450	
Introverted	55	153	
Maternal Personality			0.3061(df = 2)	0.858
Extroverted	39	110	
Middle	145	412	
Introverted	51	160	
Major Stressful Life Event #	26	56	1.7471(df = 1)	0.186
Paternal Smoking and Areca Chewing History	112	281	2.9761(df = 1)	0.085
Maternal Abortion History	51	121	1.7991(df = 1)	0.180
Use of Progestin to Prevent Threatened Abortion #	36	51	12.5141(df = 1)	0.000^*^
Use of Progestin Contraceptives at the Time of Conception	26	41	6.5871(df = 1)	0.010^*^
Prenatal Consumption of Progestin-Contaminated Seafood (g/week)			38.7781(df = 2)	0.000^*^
100–400	69	359	
400–800	92	191	
800–1,200	74	132	

* *, indicates significant difference; #, during the first trimester of pregnancy; df, degrees of freedom*.

### Multivariate logistic regression analysis for ASD risk factors

Based on the potential risk factors we identified in the case-control study from Table 5, we then conducted multivariate logistic regression analysis including those significant factors with *P* < 0.05 in χ^2^ test, and also extended to those factors that reached statistical trend level (*P* = 0.06 to 0.09, see details in Table 6). Our results showed that some factors, including maternal age < 20 years and paternal smoking and areca chewing history, had no significant effect. By contrast, the following factors: maternal age >34 years, use of progestin to prevent threatened abortion during the first trimester of pregnancy, use of progestin contraceptives at the time of conception, and prenatal consumption of progestin-contaminated seafood during the first trimester of pregnancy, had significant effects on ASD prevalence.

**Table 6:** Multivariate logistic regression analysis for impact factors on ASD prevalence.

**Impact factors**	**β**	**SE**	**Wald χ^2^**	***P* value**	**Odds ratio (95% CI)**
Maternal Age (21–34 years (Ref))			12.373	0.002*
< 20 years	0.640	0.471	1.841	0.175	1.527(0.209–2.329)
>34 years	2.605	0.775	11.913	0.001*	2.074(1.017–2.324)
Paternal Smoking and Areca Chewing History	1.317	0.778	2.867	0.090	3.733 (0.813–17.151)
Use of Progestin to Prevent Threatened Abortion	2.863	0.537	24.925	0.000*	14.631 (5.103–41.952)
Use of Progestin Contraceptives at the Time of Conception	2.756	0.371	55.234	0.000*	15.743 (7.610–32.568)
Prenatal Consumption of Progestin-Contaminated Seafood (g/week) (100–400 (Ref))			85.948	0.000*
400–800	3.583	0.395	82.187	0.000*	35.998 (16.589–78.115)
800–1,200	4.643	0.631	54.067	0.000*	103.863 (30.128–358.057)

Ref, reference; SE, standard error; *, indicates significant difference.

### Progestin exposure in water suppressed ERβ expression and its target genes in the brains of zebrafish

**Figure 1:**
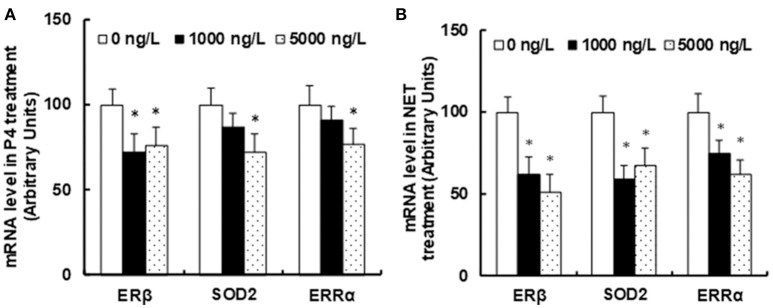
Progestin exposure in water suppresses ERβ expression and its target genes in the brains of zebrafish. Adult male zebrafish (10 months old) were exposed to 0, 500 or 1,000 ng/L of either endogenous (P4) or synthetic (NET) progestin in the water continuously for 1 month. The brains of zebrafish were isolated for the analysis of mRNA expression. **(A)** mRNA level in P4 treatment. **(B)** mRNA levels in NET treatment. *n* = 5, *P < 0.05, vs. 0 ng/L group. Results are expressed as mean ± SEM.

We investigated the potential impact of progestin contamination on seafood using zebrafish as a representative source. The zebrafish were exposed to either endogenous (P4) or synthetic (NET) progestin continuously for 1 month, and the progestin in the water was refreshed every 24 h. We first measured the stability of P4 and NET in the water after 24 h treatment. In Table 7, the measured concentrations of both P4 and NET showed no significant difference from nominated concentrations, indicating that NET and P4 were stable during the experiment process. We then measured the mRNA expression of ERβ and its target genes in the brains of treated zebrafish. In Figure 1A, P4 treatment with 1,000 and 5,000 ng/L decreased ERβ expression by 28 and 24%, respectively compared to 0 ng/L P4 control group. On the other hand, 1,000 ng/L of P4 treatment had no effect on the expression of SOD2 and ERRα, while 5,000 ng/L P4 decreased SOD2 and ERRα mRNA levels by 28 and 23%, respectively. In Figure 1B, 1,000 ng/L of NET treatment decreased mRNA expression of ERβ, SOD2 and ERRα by 38, 41, and 25%, respectively. On the other hand, 5,000ng/L of NET treatment decreased mRNA expression of ERβ, SOD2 and ERRα by 49, 33, and 38%, respectively. In addition, the effect of NET was largely not dose dependent. The statistical power was calculated and shown in Table S1. While the difference was more of a qualitative impression, our results showed that synthetic progestin NET treatment seemed to have a stronger effect on suppressing gene expression of ERβ and its target genes compared to endogenous progestin P4.

**Table 7:** The nominal and measured progestin levels in the zebrafish exposure experiments.

**Progestin**	**Exposure time (h)**	**Nominal concentration (ng/L)**		
		**0**	**1,000**	**5,000**
Progesterone (P4)	0	0	986 ± 89	4981 ± 234
	24	0	914 ± 77	4895 ± 312
	Average	0	966 ± 82	4937 ± 287
Norethindrone (NET)	0	0	1021 ± 76	4982 ± 289
	24	0	987 ± 81	5102 ± 325
	Average	0	1002 ± 84	5045 ± 269

### Prenatal consumption of progestin-contaminated seafood suppresses ERβ expression and its target genes in the amygdala from offspring in rats

**Figure 2:**
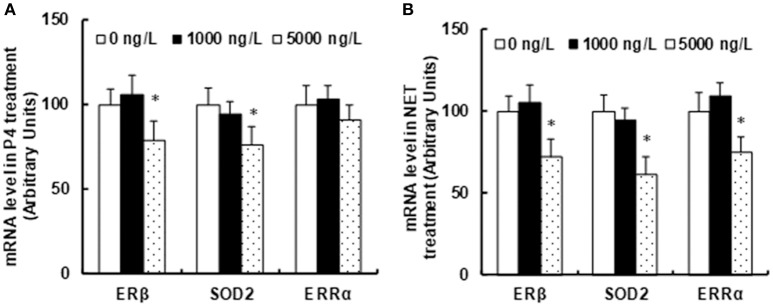
Prenatal consumption of progestin-contaminated seafood suppresses ERβ expression and its target genes in the amygdala of offspring in rats. Three months old pregnant dams were fed with progestin (P4 or NET)-contaminated seafood continuously for 21 days until pup delivery. The amygdala from both male and female offspring at 10 weeks of age were isolated for the analysis of mRNA expression. **(A)** mRNA level in P4 treatment. **(B)** mRNA levels in NET treatment. *n* = 5,*P < 0.05, vs. 0 ng/L group. Results are expressed as mean ± SEM.

We measured the potential effect of prenatal progestin exposure on the offspring. The progestin-exposed zebrafish were used to feed 3 months old pregnant dams continuously for 21 days until pup delivery. The amygdala from both male and female offspring at 10 weeks old were isolated for the analysis of mRNA expression. We first measured the effect of endogenous progestin (P4)-contaminated seafood on gene expression. In Figure 2A, the prenatal exposure of 1,000 ng/L P4-treated seafood had no effect on gene expression, while 5,000 ng/L P4-treated seafood suppressed the expression of ERβ and SOD2 by 21 and 24%, respectively, and there was no effect on ERRα expression. We then measured the effect of synthetic progestin (NET)-contaminated seafood on gene expression. In Figure 2B, prenatal exposure of 1,000 ng/L NET-treated seafood had no effect on gene expression, while 5,000 ng/L NET-treated seafood suppressed the expression of ERβ, SOD2 and ERRα by 28, 39, and 25%, respectively. The statistical power was calculated and shown in Table S1. Our results indicate that synthetic progestin (NET)-contaminated seafood had a stronger effect than endogenous progestin (P4)-contaminated seafood on the suppression of ERβ and its target genes.

### Prenatal consumption of progestin-contaminated seafood induces autism-like behavior in offspring

**Figure 3:**
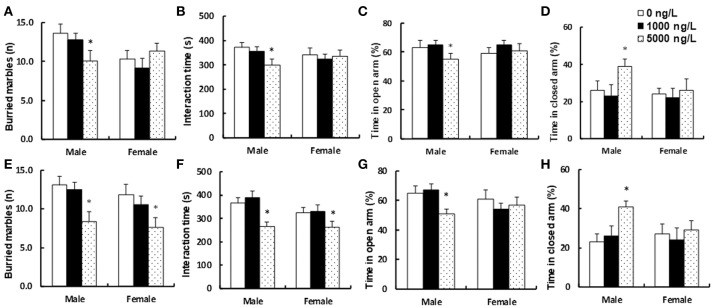
Prenatal consumption of progestin-contaminated seafood induces autism-like behavior in offspring. Three months old pregnant dams were fed with progestin (P4 or NET)-contaminated seafood continuously for 21 days until pup delivery. Both male and female offspring were used for autism-like behavior tests at 10 weeks old. (**A–D**) Autism-like behavior tests for endogenous progestin (P4) treated offspring. **(A)** Buried marbles tests, *n* = 8. **(B)** Interaction time, *n* = 9. **(C,D)** Time spent in open **(C)** and closed **(D)** arms in EPM test, *n* = 8. **(E–H)** Autism-like behavior tests for synthetic progestin (NET) treated offspring. **(E)** Buried marbles tests, *n* = 9. **(F)** Interaction time, *n* = 9. **(G,H)** Time spent in open **(G)** and closed **(H)** arms in EPM test, *n* = 9, *P < 0.05, vs. 0 ng/L group. Results are expressed as mean ± SEM.

We measured the potential effect of prenatal exposure to progestin-contaminated seafood on autism-like behavior in offspring. Three months old pregnant dams were fed with progestin (NET)-contaminated seafood zebrafish continuously for 21 days until pup delivery, and both male and female offspring were used for autism-like behavior tests at 10 weeks of age. We first measured the effect of endogenous progestin (P4)-contaminated seafood on autism-like behavior. In Figures 3A-D, all of the dosages of P4 treatment had no effect on female offspring, and 1,000 ng/L P4 had no effect on male offspring, while rats given 5,000 ng/L P4 treatment showed 26% lower numbers of buried marbles (*n* = 8, see Figure 3A), 20% less social interaction time (*n* = 9, see Figure 3B), and spent 13% less time in the open arm (*n* = 8, see Figure 3C) and 150% more time in the closed arm (*n* = 8, see Figure 3D) in the EPM (elevated plus maze) test compared to the 0 ng/L P4 treatment. In Figure 3A, Sex had a significant effect (*F* = 33.13, *P* < 0.000); P4 treatment had a significant effect (*F* = 5.77, *P* = 0.006); and there was a significant interaction between Sex and Treatment in the buried marbles test (*F* = 23.66, *P* < 0.000). In Figure 3B, Sex had a significant effect (*F* = 6.27, *P* = 0.016); P4 treatment had a significant effect (*F* = 3.88, *P* = 0.027); and there was a significant interaction between Sex and Treatment in interaction time (*F* = 4.77, *P* = 0.013). In Figure 3C, both Sex and P4 treatment had no significant effect, and there was no significant interaction between Sex and Treatment in Time in open arm. In Figure 3D, Sex had a significant effect (*F* = 15.07, *P* = 0.000); P4 treatment had a significant effect (*F* = 19.17, *P* < 0.000); and there was a significant interaction between Sex and Treatment in Time in closed arm (*F* = 7.10, *P* = 0.002). We then measured the effect of synthetic progestin (NET)-contaminated seafood on autism-like behavior. In Figures 3E–H, 1,000 ng/L NET treatment had no effect on either male or female offspring. In male offspring, rats given 5,000 ng/L NET treatment resulted in 36% lower numbers of buried marbles (*n* = 9, see Figure 3E), 28% less social interaction time (*n* = 9, see Figure 3F), and spent 22% less time in the open arm (*n* = 9, see Figure 3G) and 164% more time in the closed arm (*n* = 9, see Figure 3H) in the EPM (elevated plus maze) test compared to the 0ng/L NET treatment. On the other hand, in female offspring, 5,000 ng/L NET treatment resulted in 36% lower numbers of buried marbles (see Figure 3E), 19% less social interaction time (see Figure 3F), and had no effect on the EPM test (see Figures 3G,H) compared to 0 ng/L NET treatment. In Figure 3E, Sex had no significant effect (*F* = 0.86, *P* = 0.358); NET treatment had a significant effect (*F* = 5.35, *P* = 0.008); and there was no significant interaction between Sex and Treatment in Buried marbles tests (*F* = 0.45, *P* = 0.638). In Figure 3F, Sex had no significant effect (*F* = 3.74, *P* = 0.060); NET treatment had a significant effect (*F* = 11.50, *P* < 0.000); and there was no significant interaction between Sex and Treatment in Interaction time (*F* = 2.85, *P* = 0.069). In Figure 3G, Sex had a significant effect (*F* = 8.48, *P* = 0.006); NET treatment had a significant effect (*F* = 3.84, *P* < 0.029); and there was no significant interaction between Sex and Treatment in Time in open arm (*F* = 0.67, *P* = 0.517). In Figure 3H, Sex had a significant effect (*F* = 5.51, *P* = 0.023); NET treatment had a significant effect (*F* = 4.21, *P* = 0.021); and there was a significant interaction between Sex and Treatment in Time in closed arm (*F* = 16.46, *P* < 0.000). The detailed statistical information is shown in Data S1 and the statistical power was calculated and shown in Table S2. Our results indicate that prenatal exposure to NET-contaminated seafood had a stronger effect on inducing autism-like behavior in offspring compared to P4-contaminated seafood. In addition, female offspring were less responsive to progestin-contaminated seafood-induced autism-like behavior than male offspring.

## Discussion

In this study, our epidemiological data show that prenatal progestin exposure was a strong impact factor for ASD prevalence. Our further *in vivo* experiments in rats show that prenatal exposure to progestin-contaminated seafood induced autism-like behavior in offspring, which further demonstrated that prenatal progestin exposure is associated with ASD prevalence.

### ASD prevalence in hainan province of china

We show that the ASD prevalence rate in Hainan province was 0.62%, which is lower than the ASD prevalence rate of 0.83% in Shanghai (43). Furthermore, it is much lower than the latest reported ASD prevalence rate of 1:68 in the USA (12, 44). It is possible that since Hainan province is an independent island separated from mainland China, there is much less pollution from heavy industry, so that most of the seafood in Hainan are natural products instead of commercial products fed with progestin. On the other hand, much of the seafood in Shanghai is commercial seafood with progestin contamination. Furthermore, we speculate that in the USA, wide use of progestin-containing contraceptives may be an important factor that results in higher ASD prevalence. In addition, our epidemiological study in Hainan province shows that ASD prevalence in economically developed regions was higher than in less-developed regions. This may be partly because mothers in economically developed regions were more likely to ingest progestin-contaminated seafood, or to take progestin to prevent threatened abortion during their pregnancy as a result of better medical and living resources. On the other hand, many ASD positive cases might be ignored or were not brought to the attention of the parents due to lack of medical resources, which potentially could contribute to the lower rates of diagnosed ASD prevalence in the less developed regions.

### Potential impacts of gender and age in ASD prevalence

Our data show that ASD prevalence in Hainan was a significant gender bias with a male/female ratio of 5.8/1 compared to the rate of 4:1 as generally reported in the USA (12, 44). The reason was likely that the population of Hainan has a significant gender bias with a boy:girl ratio of 1.2:1, which potentially could contribute to a higher ASD prevalence ratio in boys. Furthermore, we have previously reported that prenatal progestin exposure suppresses ERβ expression in the brain and triggers autism-like behavior in rats (19). Interestingly, the basal expression of ERβ in females was significantly higher than in males. In addition, ERβ activity in females was significantly higher than in males due to the existence of endogenous estradiol. In this case, females with higher ERβ activity was more resistant to progestin-induced ERβ suppression and subsequent cell damage triggered by oxidative stress (45) and dysfunction of mitochondria and lipid metabolism (46, 47). Relatively higher ERβ expression and activity in the brains of females can be a potential reason why females have lower ASD prevalence. In addition, our data show that ASD prevalence in Hainan was significantly increased with increasing age. This may suggest that many ASD children were not diagnosed properly in time, or that symptoms were not brought to the attention of their parents during their early ages, or symptoms might simply take time to develop in severity.

### Potential impact factors on ASD prevalence

In this study, we have investigated many potential impact factors for ASD prevalence. Based on our present sample, we did not find potential effects on ASD prevalence for the factors of family history of mental diseases, paternal age, paternal personality or maternal personality, but cannot exclude such factors entirely. For instance, there could be insufficient variation in some of the factors to allow for the identification of their contributions. Furthermore, we found small effects on ASD prevalence for the risk factors of pre-term birth (< 37 weeks), birth asphyxia, maternal age, major stressful life event during the first trimester of pregnancy, paternal smoking and areca chewing history, and maternal abortion history. Pre-term birth and maternal abortion history are usually associated with the use of progestin to prevent threatened abortion (48, 49), and birth asphyxia is usually directly associated with brain damage due to the hypoxic condition (50). Maternal age (< 20 years) is usually associated with the use of progestin contraceptives, and like the factor of a major stressful life event, it is also associated with high pressure-induced hormone disorder, while the factor of paternal smoking and areca chewing history is usually associated with direct damage of semen from smoking and areca consumption (51, 52).

### Significant impact of prenatal progestin exposure on ASD prevalence

Our data show that the following factors have significant impacts on ASD prevalence (with OR>1.60): maternal age (>34 years), use of progestin to prevent threatened abortion, use of progestin contraceptives at the time of conception, and prenatal consumption of progestin-contaminated seafood during the first trimester of pregnancy. All the above factors are associated with prenatal progestin exposure. Our survey in Hainan province shows that almost all the mothers >34 years old (66 from 69 in total) were instructed to take either endogenous (P4, 7 from 66 cases) or synthetic progestin (59 from 66 cases) in order to prevent potential threatened abortion during their first trimester of pregnancy. It has also been reported that the activity of synthetic progestin has much stronger (higher than 10 times) effect than endogenous progestin (P4). This suggests that the maternal age itself may not be the direct factor. Instead progestin intake plays a critical role in ASD development; another detailed investigation on this specific issue is still in process. On the other hand, some other factors, such as epigenetic factors that are unrelated to progestin intake, may also explain why maternal age (>34 years) was strongly associated with ASD prevalence. Furthermore, progestin may modulate the expression of some other receptors (e.g., glucocorticoid receptor), and potentially contribute to ASD development, while our investigation showed that progestin did not change the expression of glucocorticoid receptor in the brain, even though it has some effects on the vascular system (53). During the commercial feeding process in China, seafood (including fish and lobsters) are fed purposely with progestin-containing contraceptives to prevent female pregnancy in order to ensure fresh and tasty seafood meat. We defined progestin-contaminated seafood as being any fish, lobsters, etc. that were fed with progestin-containing contraceptives, and consumption of natural seafood was excluded. In this study, seafood was considered to be the major source of progestin-contaminated food because seafood was usually the first choice of the mothers during their pregnancy due to its good nutrition. However, of course, any other food that is contaminated with progestin could potentially contribute to ASD prevalence as well. The quantitation for maternal consumption of progestin-contaminated seafood was separated into 3 scales as following: (a) 100–400 g/week (0–1 serving per week); (b) 400–800 g/week (2–3 servings per week); (c) 800–1,200 g/week (4–7 servings per week) (54). Our results show that maternal consumption of 800–1,200 g/week during the first trimester of pregnancy was strongly associated with ASD prevalence, indicating that prenatal progestin exposure may be associated with ASD development.

### Prenatal consumption of progestin-contaminated seafood induces autism-like behavior in offspring from rats

We have reported previously that prenatal progestin exposure can induce autism-like behavior in the offspring of rats (19). Here we wanted to further test the hypothesis that prenatal consumption of progestin-contaminated seafood would induce autism-like behavior in rats as well. The zebrafish was first exposed to large amounts of either endogenous (P4) or synthetic (NET) progestin to mimic the chronic exposure of seafood to progestin contamination during the commercial feeding process. In this study, the amount of progestin in the seafood was not measured because it was metabolized soon, not measurable using HPLC or any better alternative methods. We show that progestin exposure induced significant ERβ suppression in the brains of zebrafish. This is consistent with our previous finding (19), indicating a successful progestin exposure in seafood. Furthermore, the progestin-contaminated zebrafish were used to feed pregnant dams, and autism-like behavior was observed in addition to ERβ suppression in the brain in offspring. This further demonstrated that prenatal consumption of progestin-contaminated seafood can induce autism-like behavior in offspring, and indicates that prenatal progestin exposure is associated with ASD development.

## Conclusions

Taken together, our epidemiology study showed that prenatal progestin exposure was strongly associated with ASD prevalence, and the experiments in rats showed that prenatal consumption of progestin-contaminated seafood induced autism-like behavior. We conclude that prenatal progestin exposure may be associated with ASD development.

## Author contributions

PY wrote the paper. PY, HC, and XH designed, interpreted the experiments. ML, WgX and XH performed the rat surgery and social behavior testing. XG, XW, YW, JL, and HC performed gene analysis and statistical analysis. LL, XL, CL, YH, LZ, WX, and ZW performed ASD epidemiology study. LL, ML, and JL performed the remaining experiments. All authors read, edited, and approved the final manuscript.

### Conflict of interest statement

Authors including XG, XW, YW, and HC, were employed by company Guangzhou SALIAI Stem Cell Science and Technology Co. LTD. The remaining authors declare that the research was conducted in the absence of any commercial or financial relationships that could be construed as a potential conflict of interest.

## Abbreviations

ASD: autism spectrum disorder
EPM: elevated plus maze
ERβ: estrogen receptor β
ERRα: estrogen-related receptor α
MBT: marbles burying test
NET: norethindrone
P4: progesterone
SI: social interaction
SOD2: mitochondrial superoxide dismutase
TD: typically developing.
