# Supplementary material for: Prenatal Progestin Exposure Is Associated With Autism Spectrum Disorders
Source: Front Psychiatry. 2018 Nov 19;9:611. doi: 10.3389/fpsyt.2018.00611 (PMC6252360; doi:10.3389/fpsyt.2018.00611)
Supplement: Supplementary file 1 [file Table_1.DOCX]

**Prenatal Progestin Exposure is Associated with Autism Spectrum Disorders**

Ling Li^1,^*, Min Li^2,^*, Jianping Lu^3,^*, Xiaohu Ge^4^, Weiguo Xie^2^, Zichen Wang^3^, Xiaoling Li^1^, Chao Li^1^, Xiaoyan Wang^4^, Yan Han^1^, Yifei Wang^4^, Liyan Zhong^1^, Wei Xiang^1^, Xiaodong Huang^2,#^, Haijia Chen^4,#^, Paul Yao^1,2,3,#^

**Supplemental Data**

**Table S1. Statistical power for Figure 1 and 2**

| Statistical Power | Factors | | |
| --- | --- | --- | --- |
|  | ERβ | SOD2 | ERRα |
| Figure 1a | 0.970 | 0.962 | 0.925 |
| Figure 1b | 0.999 | 0.996 | 0.988 |
| Figure 2a | 0.900 | 0.802 | 0.768 |
| Figure 2b | 0.984 | 0.997 | 0.971 |

**Table S2. Statistical power for Figure 3**

| Statistical Power | Factors | |
| --- | --- | --- |
|  | Sex | Treatment |
| Figure 3a | 0.999 | 0.702 |
| Figure 3b | 0.668 | 0.999 |
| Figure 3c | 0.556 | 0.999 |
| Figure 3d | 0.874 | 0.996 |
| Figure 3e | 0.994 | 0.999 |
| Figure 3f | 0.997 | 0.999 |
| Figure 3g | 0.710 | 0.993 |
| Figure 3h | 0.955 | 0.989 |

**Data S1. The detailed statistical information for Figure 3.** A factorial design ANOVA was used for the analysis of the basic experimental design follows a full-factorial 3 ˣ 2 (prenatal treatment ˣ sex) design study, and the Fisher’s Least Significant Difference (LSD) test was conducted for multiple comparisons when there was a significant effect on prenatal treatment.

# Detailed statistical information for Figure 3a

Table S1. The buried marbles test for P4 treated offspring (Mean ± SD, n=8)

| Treatment | Sex | | Total |
| --- | --- | --- | --- |
|  | Male | Female |  |
| 0 ng/L | 13.6±1.2 | 10.3±1.1 | 12.0±2.1 |
| 1000 ng/L | 12.8±0.8 | 9.1±1.3 | 10.9±2.0 |
| 5000 ng/L | 10.1±1.3 | 11.3±1.0 | 10.7±1.3 |
| Total | 12.2±1.9 | 10.3±1.4 | 11.2±1.9 |

| Table S2. ANOVA of Sex and Treatment for the buried marbles tests | | | | |
| --- | --- | --- | --- | --- |
| Source | ANOVA SS | Mean Square | F | P |
| Sex | 42.771 | 45.771 | 33.13 | <0.000* |
| Treatment | 14.9050 | 7.453 | 5.77 | 0.006* |
| Sex × Treatment | 61.087 | 30.543 | 23.66 | <0.000* |

Conclusion: 1. Sex of rats had a significant effect on the buried marbles tests; 2. P4 treatment had a significant effect on the buried marbles tests; 3. There was a significant interaction between Sex and Treatment in the buried marbles tests. In male rats, when the Treatment was 1000 ng/L and 5000 ng/L, the buried marbles tests score were higher; 4. The LSD multiple comparison revealed that the mean value of 0ng/L P4 is larger than 1000ng/L P4 and 5000 ng/L P4.

# Detailed statistical information for Figure 3b

Table S3. Social interaction time(s) for P4 treated offspring (Mean ± SD, n=9)

| Treatment | Sex | | Total |
| --- | --- | --- | --- |
|  | Male | Female |  |
| 0 ng/L | 371±21 | 341±27 | 356±25 |
| 1000 ng/L | 356±19 | 325±19 | 340±25 |
| 5000 ng/L | 298±25 | 336±24 | 317±30 |
| Total | 342±37 | 334±23 | 338±31 |

| Table S4. ANOVA of Sex and Treatment for Interaction time | | | | |
| --- | --- | --- | --- | --- |
| Source | ANOVA SS | Mean Square | F | P |
| Sex | 4401.824 | 4401.824 | 6.27 | 0.016* |
| Treatment | 5446.301 | 2723.151 | 3.88 | 0.027* |
| Sex × Treatment | 6691.673 | 3345.836 | 4.77 | 0.013* |

Conclusion: 1. Sex of rats had a significant effect in interaction time, the male rats had a longer interaction time; 2. Treatment had a significant effect for interaction time; 3. There was a significant interaction between Sex and Treatment in interaction time. 4. The LSD multiple comparison revealed that the mean value of 5000ng/L P4 is less than 1000ng/L P4 and 0 ng/L P4.

# Detailed statistical information for Figure 3c

Table S5. Social interaction time (s) for P4 treated offspring (Mean ± SD, n=9)

| Treatment | Sex | | Total |
| --- | --- | --- | --- |
|  | Male | Female |  |
| 0 ng/L | 63±5 | 59±4 | 61±5 |
| 1000 ng/L | 65±3 | 65±3 | 65±3 |
| 5000 ng/L | 55±4 | 61±5 | 58±6 |
| Total | 61±6 | 62±5 | 61±5 |

| Table S6. ANOVA of Sex and Treatment for Time in open arm | | | | |
| --- | --- | --- | --- | --- |
| Source | ANOVA SS | Mean Square | F | P |
| Sex | 892052.229 | 892052.229 | 0.95 | 0.335 |
| Treatment | 1807991.137 | 903995.598 | 0.96 | 0.390 |
| Sex × Treatment | 1879710.939 | 939855.470 | 1.00 | 0.376 |

Conclusion: Variance analysis of factorial design revealed no significant effect of Sex and Treatment, and there was no significant Interaction between Sex and Treatment.

# Detailed statistical information for Figure 3d

Table S7. Time in closed arm (%) for P4 treated offspring (Mean ± SD, n=8)

| Treatment | Sex | | Total |
| --- | --- | --- | --- |
|  | Male | Female |  |
| 0 ng/L | 26±5 | 24±3 | 25±4 |
| 1000 ng/L | 23±6 | 22±5 | 23±6 |
| 5000 ng/L | 39±4 | 26±6 | 33±8 |
| Total | 29±9 | 24±5 | 27±7 |

| Table S8. ANOVA of Sex and Treatment for Time in closed arm | | | | |
| --- | --- | --- | --- | --- |
| Source | ANOVA SS | Mean Square | F | P |
| Sex | 370.557 | 370.557 | 15.07 | 0.000* |
| Treatment | 343.039 | 471.520 | 19.17 | <0.000* |
| Sex × Treatment | 349.216 | 174.609 | 7.10 | 0.002* |

Conclusion: 1. Sex of rats had a significant effect for Time in closed arm, the male rats stayed longer in closed arm; 2. Treatment had a significant effect for Time in closed arm; 3. There was a significant Interaction between Sex and Treatment for Time in closed arm.; 4. The LSD multiple comparisons revealed that the mean value of 5000ng/L P4 is larger than 1000ng/L P4 and 0ng/L P4.

# Detailed statistical information for Figure 3e

Table S9. Buried marbles tests(n) for NET treated offspring (Mean ± SD, n=9)

| Treatment | Sex | | Total |
| --- | --- | --- | --- |
|  | Male | Female |  |
| 0 ng/L | 13.1±1.1 | 11.8±1.4 | 12.4±1.2 |
| 1000 ng/L | 12.5±0.9 | 10.6±0.9 | 11.6±1.4 |
| 5000 ng/L | 8.4±1.2 | 7.6±1.3 | 8.0±1.3 |
| Total | 11.3±2.3 | 10.0±2.1 | 10.7±2.3 |

| Table S10. ANOVA of Sex and Treatment for Buried marbles tests | | | | |
| --- | --- | --- | --- | --- |
| Source | ANOVA SS | Mean Square | F | P |
| Sex | 3.998 | 3.998 | 0.86 | 0.358 |
| Treatment | 49.759 | 24.879 | 5.35 | 0.008* |
| Sex × Treatment | 4.224 | 2.112 | 0.45 | 0.638 |

Conclusion:1. Variance analysis of factorial design revealed a significant effect of treatment for Time in closed arm; 2. Variance analysis of factorial design revealed no significant effect of Sex; 3. Variance analysis of factorial design revealed no significant Interaction between Sex and Treatment; 4. The LSD multiple comparisons revealed that the mean value of 5000ng/L NET was less than 1000ng/LNET and 0 ng/L NET.

# Detailed statistical information for Figure 3f

Table S11. Interaction time(s) for NET treated offspring (Mean ± SD, n=9)

| Treatment | Sex | | Total |
| --- | --- | --- | --- |
|  | Male | Female |  |
| 0 ng/L | 365±24 | 325±21 | 346±33 |
| 1000 ng/L | 389±29 | 331±27 | 360±35 |
| 5000 ng/L | 265±21 | 263±25 | 265±20 |
| Total | 340±60 | 307±37 | 324±52 |

| Table S12. ANOVA of Sex and Treatment for Interaction time | | | | |
| --- | --- | --- | --- | --- |
| Source | ANOVA SS | Mean Square | F | P |
| Sex | 6474.005 | 6474.005 | 3.74 | 0.060 |
| Treatment | 39853.031 | 19926.515 | 11.50 | <0.000* |
| Sex × Treatment | 9865.447 | 4932.724 | 2.85 | 0.069 |

Conclusion: 1. Sex of rats had no significant effect for Interaction time; 2. Treatment had a significant effect for Interaction time; 3. There was no significant Interaction between Sex and Treatment; 4. The LSD multiple comparisons revealed that the mean value of 1000ng/L NET was larger than 0ng/LNET and 5000 ng/L NET, and the mean value of 5000ng/L NET was less than 0ng/L NET.

**Detailed statistical information for Figure 3g**

Table S13. Time in open arm (%) for NET treated offspring (Mean ± SD, n=9)

| Treatment | Sex | | Total |
| --- | --- | --- | --- |
|  | Male | Female |  |
| 0 ng/L | 65±5 | 61±6 | 63±6 |
| 1000 ng/L | 67±4 | 54±4 | 61±8 |
| 5000 ng/L | 51±3 | 57±5 | 54±5 |
| Total | 61±8 | 57±6 | 59±8 |

| Table S14. ANOVA of Sex and Treatment for Time in open arm | | | | |
| --- | --- | --- | --- | --- |
| Source | ANOVA SS | Mean Square | F | P |
| Sex | 404.549 | 404.549 | 8.48 | 0.006* |
| Treatment | 365.865 | 182.933 | 3.84 | 0.029* |
| Sex × Treatment | 63.913 | 31.956 | 0.67 | 0.517 |

Conclusion: 1. Sex of rats had a significant effect for Time in open arm; 2. Treatment had a significant effect for Time in open arm; 3. There was no significant Interaction between Sex and Treatment for Time in open arm; 4. The LSD multiple comparison revealed that the mean value of 5000ng/L NET was less than 0ng/LNET and 1000 ng/L NET.

# Detailed statistical information for Figure 3h

Table S15. Time in closed arm (%) for NET treated offspring (Mean ± SD, n=9)

| Treatment | Sex | | Total |
| --- | --- | --- | --- |
|  | Male | Female |  |
| 0 ng/L | 25±3 | 25±6 | 25±3 |
| 1000 ng/L | 27±5 | 23±3 | 25±4 |
| 5000 ng/L | 41±4 | 27±4 | 34±8 |
| Total | 31±8 | 24±4 | 28±7 |

| Table S16. ANOVA of Sex and Treatment for Time in closed arm | | | | |
| --- | --- | --- | --- | --- |
| Source | ANOVA SS | Mean Square | F | P |
| Sex | 151.063 | 152.063 | 5.51 | 0.023* |
| Treatment | 232.045 | 116.022 | 4.21 | 0.021* |
| Sex × Treatment | 908.031 | 454.015 | 16.46 | <0.000* |

Conclusion: 1. Sex of rats had a significant effect for Time in closed arm; 2. Treatment had a significant effect for Time in closed arm; 3. There was an Interaction between Sex and Treatment for Time in closed arm; 4. The LSD multiple comparison revealed that the mean value of 5000ng/L NET was larger than 0ng/LNET and 1000 ng/L NET.

**Table S1. Statistical power for Figure 1 and 2**

| Statistical Power | Factors | | |
| --- | --- | --- | --- |
|  | ERβ | SOD2 | ERRα |
| Figure 1a | 0.970 | 0.962 | 0.925 |
| Figure 1b | 0.999 | 0.996 | 0.988 |
| Figure 2a | 0.900 | 0.802 | 0.768 |
| Figure 2b | 0.984 | 0.997 | 0.971 |

**Table S2. Statistical power for Figure 3**

| Statistical Power | Factors | |
| --- | --- | --- |
|  | Sex | Treatment |
| Figure 3a | 0.999 | 0.702 |
| Figure 3b | 0.668 | 0.999 |
| Figure 3c | 0.556 | 0.999 |
| Figure 3d | 0.874 | 0.996 |
| Figure 3e | 0.994 | 0.999 |
| Figure 3f | 0.997 | 0.999 |
| Figure 3g | 0.710 | 0.993 |
| Figure 3h | 0.955 | 0.989 |
